# 2,2′-[(1,3,4-Thia­diazole-2,5-di­yl)bis­(sulfanedi­yl)]diaceto­nitrile

**DOI:** 10.1107/S1600536813032194

**Published:** 2013-11-30

**Authors:** Joel T. Mague, Mehmet Akkurt, Shaaban K. Mohamed, Ahmed M. M. El-Saghier, Mustafa R. Albayati

**Affiliations:** a Department of Chemistry, Tulane University, New Orleans, LA 70118, USA; b Department of Physics, Faculty of Sciences, Erciyes University, 38039 Kayseri, Turkey; c Chemistry and Environmental Division, Manchester Metropolitan University, Manchester M1 5GD, England; d Chemistry Department, Faculty of Science, Minia University, 61519 El-Minia, Egypt; e Department of Chemistry, Faculty of Science, Sohag University, 82524 Sohag, Egypt; f Kirkuk University, College of Science, Department of Chemistry, Kirkuk, Iraq

## Abstract

In the title compound, C_6_H_4_N_4_S_3_, the 1,3,4-thia­diazole ring is essentially planar, with an r.m.s. deviation of 0.001 Å. The two N—C—S—C torsion angles in the mol­ecule are −23.41 (15) and 0.62 (14)°. One aceto­nitrile group is above the plane of the 1,3,4-thia­diazole ring and the other is below it, indicating *syn* and *anti* orientations. In the crystal, C—H⋯N hydrogen bonds link the molecules into ribbons along [010].

## Related literature

For the broad spectrum of biological activities of thia­diazole-containing compounds, see: Padmavathi *et al.* (2009 ▶); Karegoudar *et al.* (2008 ▶); Wei *et al.* (2009 ▶); Gupta *et al.* (2009 ▶); Pattanayak *et al.* (2009 ▶); Cressier *et al.* (2009 ▶).
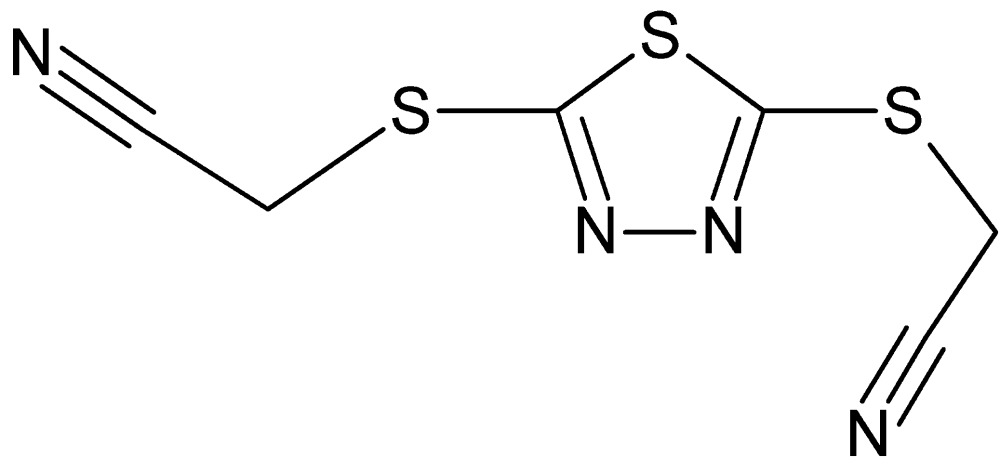


## Experimental

### 

#### Crystal data

C_6_H_4_N_4_S_3_
*M* *_r_* = 228.34
Monoclinic, 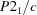

*a* = 8.5305 (7) Å
*b* = 14.2102 (11) Å
*c* = 7.8803 (6) Å
β = 104.3810 (11)°
*V* = 925.32 (13) Å^3^
*Z* = 4
Mo *K*α radiation
μ = 0.76 mm^−1^
*T* = 150 K
0.24 × 0.08 × 0.06 mm

#### Data collection

Bruker SMART APEX CCD diffractometer
Absorption correction: multi-scan (*SADABS*; Bruker, 2013 ▶) *T*_min_ = 0.82, *T*_max_ = 0.96
16625 measured reflections
2450 independent reflections
2155 reflections with *I* > 2σ(*I*)
*R*_int_ = 0.040

#### Refinement

*R*[*F* ^2^ > 2σ(*F* ^2^)] = 0.029
*wR*(*F* ^2^) = 0.074
*S* = 1.05
2450 reflections
118 parameters
H-atom parameters constrained
Δρ_max_ = 0.56 e Å^−3^
Δρ_min_ = −0.24 e Å^−3^

### 

Data collection: *APEX2* (Bruker, 2013 ▶); cell refinement: *SAINT* (Bruker, 2013 ▶); data reduction: *SAINT*; program(s) used to solve structure: *SHELXT* (Sheldrick, 2008) ▶; program(s) used to refine structure: *SHELXL2013* (Sheldrick, 2008) ▶; molecular graphics: *ORTEP-3 for Windows* (Farrugia, 2012 ▶); software used to prepare material for publication: *WinGX* (Farrugia, 2012 ▶) and *PLATON* (Spek, 2009 ▶).

## Supplementary Material

Crystal structure: contains datablock(s) global, I. DOI: 10.1107/S1600536813032194/hg5363sup1.cif

Structure factors: contains datablock(s) I. DOI: 10.1107/S1600536813032194/hg5363Isup2.hkl

Supplementary material file. DOI: 10.1107/S1600536813032194/hg5363Isup3.cml

Additional supplementary materials:  crystallographic information; 3D view; checkCIF report

## Figures and Tables

**Table 1:** Hydrogen-bond geometry (Å, °)

*D*—H⋯*A*	*D*—H	H⋯*A*	*D*⋯*A*	*D*—H⋯*A*
C3—H3*A*⋯N1^i^	0.99	2.60	3.407 (2)	139
C5—H5*B*⋯N3^ii^	0.99	2.35	3.267 (2)	153

Symmetry codes: (i) 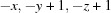; (ii) 
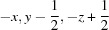
.

**Fig. 1.**
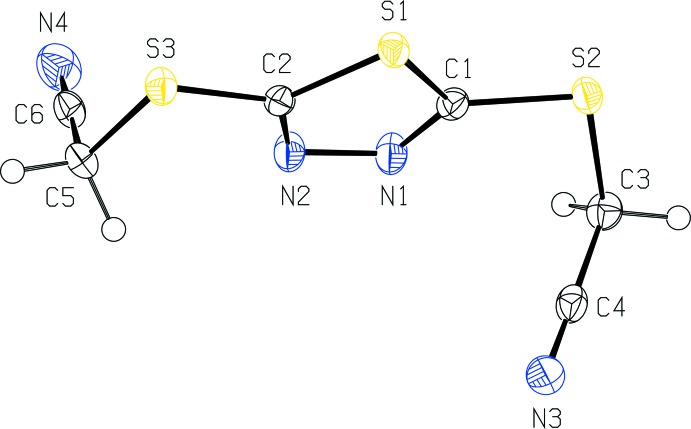
Perspective view of the title molecule with 50% probability displacement ellipsoids.

**Fig. 2.**
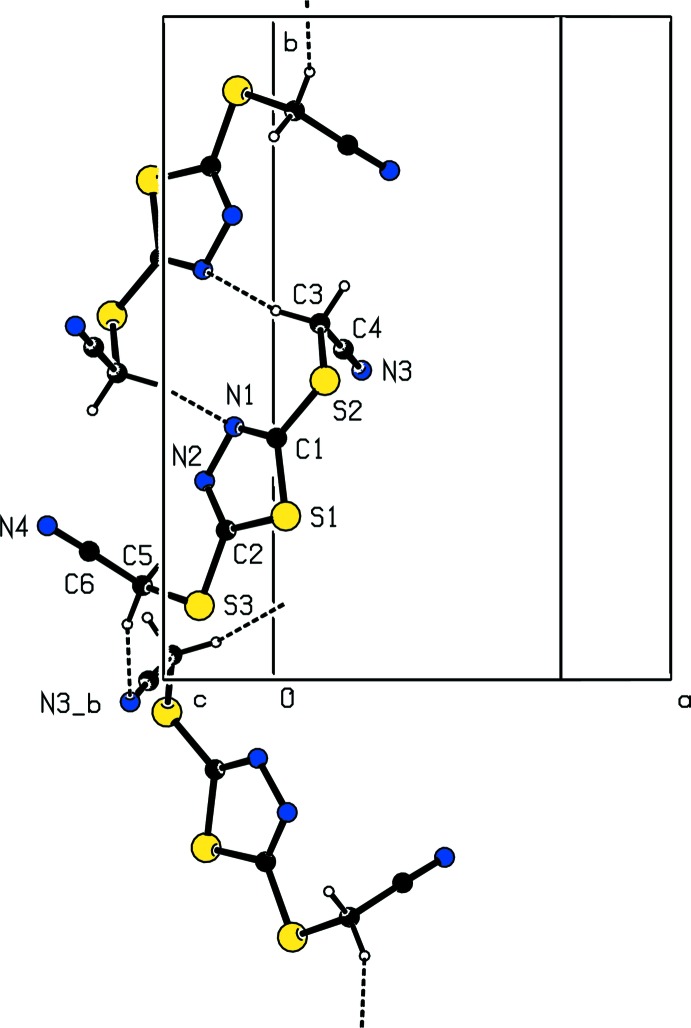
The hydrogen bonding (dashed lines) viewed along the b-axis of the title compound. [Symmetry code: (b) 1 + x, ½ - y, ½ + z].

## supplementary crystallographic information

### 1. Comment

Thiadiazole acts as a constrained pharmacophore. It is the core structure of several medicinal drugs such as acetazolamide, atibeprone, tebuthiuron and methazolamide. In addition, thiadiazole containing compounds have a wide spectrum of biological activities such as antimicrobial (Padmavathi *et al*., 2009), antiinflammatory (Karegoudar *et al.*, 2008), anticancer (Wei *et al.*, 2009), anticonvulsant (Gupta *et al.*, 2009), antidepressant (Pattanayak *et al.*, 2009), and antioxidant (Cressier *et al.*, 2009). Based on such facts, the title compound has been synthesized in our lab as a precurser for further study.

The 1,3,4-thiadiazole ring (S1/N1/N2/C1/C2) of the title compound, (I, Fig. 1), is essentially planar [r.m.s deviation = 0.001 Å]. The N1–C1–S2–C3 and N2–C2–S3–C5 torsion angles in (I) are 23.41 (15) and -0.62 (14)°, respectively. The two acetonitrile groups [–C3(H_2_)–C4≡N3 and –C5(H_2_)–C6≡N4] of (I) are above and below the plane of the 1,3,4-thiadiazole ring, indicating *syn-* and *anti-* orientations, respectively.

In the crystal, molecules are linked by intermolecular C—H···N hydrogen bonds to form ribbons along the *b*-axis (Table 1, Fig. 2).

### 2. Experimental

A mixture of 1,3,4-thiadiazolidine-2,5-dithione (150 mg, 1 mmol), chloroacetonitrile (149 mg, 2 mmol), sodium acetate (36 mg, 0.5 mmol) in 30 ml e thanol was refluxed for 4 h. The reaction mixture was allowed to cool to room temperature to afford the solid product which was filtered off under vacuum, dried and recrystallized from ethanol. Pure single crystals were prepared by slow evaporation of an ethanolic solution of the title compound in air over 24 h. M·P. 396 K.

### 3. Refinement

The methylene H atoms were positioned geometrically and refined by using a riding model with C—H = 0.99 Å and, with *U*_iso_(H) = 1.2*U*_iso_(C).

### Crystal data

**Table:**

C_6_H_4_N_4_S_3_	*F*(000) = 464
*M**_r_* = 228.34	*D*_x_ = 1.639 Mg m^−^^3^
Monoclinic, *P*2_1_/*c*	Mo *K*α radiation, λ = 0.71073 Å
Hall symbol: -P 2ybc	Cell parameters from 9961 reflections
*a* = 8.5305 (7) Å	θ = 2.9–29.1°
*b* = 14.2102 (11) Å	µ = 0.76 mm^−^^1^
*c* = 7.8803 (6) Å	*T* = 150 K
β = 104.3810 (11)°	Column, pale gold
*V* = 925.32 (13) Å^3^	0.24 × 0.08 × 0.06 mm
*Z* = 4	

### Data collection

**Table:**

Bruker SMART APEX CCD diffractometer	2450 independent reflections
Radiation source: fine-focus sealed tube	2155 reflections with *I* > 2σ(*I*)
Graphite monochromator	*R*_int_ = 0.040
Detector resolution: 8.3660 pixels mm^-1^	θ_max_ = 29.1°, θ_min_ = 2.5°
φ and ω scans	*h* = −11→11
Absorption correction: multi-scan (*SADABS*; Bruker, 2013)	*k* = −19→19
*T*_min_ = 0.82, *T*_max_ = 0.96	*l* = −10→10
16625 measured reflections	

### Refinement

**Table:**

Refinement on *F*^2^	0 restraints
Least-squares matrix: full	Hydrogen site location: inferred from neighbouring sites
*R*[*F*^2^ > 2σ(*F*^2^)] = 0.029	H-atom parameters constrained
*wR*(*F*^2^) = 0.074	W = 1/[Σ^2^(*F*O^2^) + (0.0348*P*)^2^ + 0.4414*P*] Where *P* = (*F*O^2^ + 2*F*C^2^)/3
*S* = 1.05	(Δ/σ)_max_ < 0.001
2450 reflections	Δρ_max_ = 0.56 e Å^−^^3^
118 parameters	Δρ_min_ = −0.24 e Å^−^^3^

### Special details

**Table:**

Geometry. Bond distances, angles *etc*. have been calculated using the rounded fractional coordinates. All su's are estimated from the variances of the (full) variance-covariance matrix. The cell e.s.d.'s are taken into account in the estimation of distances, angles and torsion angles
Refinement. Refinement on *F*^2^ for ALL reflections except those flagged by the user for potential systematic errors. Weighted *R*-factors *wR* and all goodnesses of fit *S* are based on *F*^2^, conventional *R*-factors *R* are based on *F*, with *F* set to zero for negative *F*^2^. The observed criterion of *F*^2^ > σ(*F*^2^) is used only for calculating -*R*-factor-obs *etc*. and is not relevant to the choice of reflections for refinement. *R*-factors based on *F*^2^ are statistically about twice as large as those based on *F*, and *R*-factors based on ALL data will be even larger.

### Fractional atomic coordinates and isotropic or equivalent isotropic displacement parameters (Å^2^)

**Table:**

	*x*	*y*	*z*	*U*_iso_*/*U*_eq_	
S1	0.24557 (4)	0.24679 (2)	0.77612 (5)	0.0199 (1)	
S2	0.35261 (4)	0.45127 (3)	0.80614 (5)	0.0225 (1)	
S3	−0.02136 (4)	0.11179 (3)	0.59651 (5)	0.0217 (1)	
N1	0.07063 (16)	0.38153 (9)	0.61222 (18)	0.0246 (4)	
N2	−0.01784 (15)	0.29982 (9)	0.56140 (18)	0.0232 (4)	
N3	0.31454 (18)	0.46664 (10)	0.33409 (18)	0.0286 (4)	
N4	−0.40861 (18)	0.23221 (12)	0.5806 (2)	0.0370 (5)	
C1	0.20800 (17)	0.36440 (10)	0.72254 (19)	0.0188 (4)	
C2	0.05731 (17)	0.22555 (10)	0.63639 (18)	0.0178 (4)	
C3	0.29143 (18)	0.53720 (10)	0.6312 (2)	0.0223 (4)	
C4	0.30417 (18)	0.49844 (10)	0.4636 (2)	0.0222 (4)	
C5	−0.20837 (18)	0.14204 (11)	0.4383 (2)	0.0240 (4)	
C6	−0.32061 (18)	0.19370 (11)	0.5168 (2)	0.0247 (4)	
H3A	0.17830	0.55670	0.62240	0.0270*	
H3B	0.36100	0.59370	0.65920	0.0270*	
H5A	−0.18280	0.18080	0.34420	0.0290*	
H5B	−0.26140	0.08360	0.38420	0.0290*	

### Atomic displacement parameters (Å^2^)

**Table:**

	*U*^11^	*U*^22^	*U*^33^	*U*^12^	*U*^13^	*U*^23^
S1	0.0170 (2)	0.0195 (2)	0.0210 (2)	−0.0004 (1)	0.0001 (1)	0.0025 (1)
S2	0.0207 (2)	0.0212 (2)	0.0224 (2)	−0.0041 (1)	−0.0007 (1)	0.0015 (1)
S3	0.0205 (2)	0.0189 (2)	0.0243 (2)	−0.0020 (1)	0.0029 (1)	−0.0006 (1)
N1	0.0195 (6)	0.0210 (6)	0.0302 (7)	−0.0028 (5)	0.0006 (5)	0.0037 (5)
N2	0.0185 (6)	0.0212 (6)	0.0274 (7)	−0.0031 (5)	0.0012 (5)	0.0025 (5)
N3	0.0310 (7)	0.0273 (7)	0.0261 (7)	−0.0034 (6)	0.0042 (6)	0.0016 (6)
N4	0.0244 (7)	0.0490 (9)	0.0352 (8)	0.0019 (7)	0.0031 (6)	−0.0032 (7)
C1	0.0195 (7)	0.0175 (6)	0.0194 (7)	−0.0010 (5)	0.0050 (5)	0.0017 (5)
C2	0.0156 (6)	0.0217 (7)	0.0164 (6)	−0.0014 (5)	0.0043 (5)	0.0004 (5)
C3	0.0232 (7)	0.0173 (7)	0.0260 (8)	0.0014 (5)	0.0051 (6)	0.0023 (6)
C4	0.0202 (7)	0.0180 (7)	0.0265 (8)	−0.0026 (5)	0.0025 (6)	0.0041 (6)
C5	0.0233 (7)	0.0272 (8)	0.0187 (7)	−0.0049 (6)	0.0000 (5)	−0.0028 (6)
C6	0.0187 (7)	0.0299 (8)	0.0215 (7)	−0.0047 (6)	−0.0026 (6)	0.0009 (6)

### Geometric parameters (Å, º)

**Table:**

S1—C1	1.7340 (15)	N3—C4	1.140 (2)
S1—C2	1.7333 (15)	N4—C6	1.142 (2)
S2—C1	1.7538 (15)	C3—C4	1.460 (2)
S2—C3	1.8184 (15)	C5—C6	1.460 (2)
S3—C2	1.7486 (15)	C3—H3A	0.9900
S3—C5	1.8155 (16)	C3—H3B	0.9900
N1—N2	1.3885 (19)	C5—H5A	0.9900
N1—C1	1.297 (2)	C5—H5B	0.9900
N2—C2	1.2984 (19)		
			
C1—S1—C2	85.82 (7)	S3—C5—C6	112.64 (11)
C1—S2—C3	98.35 (7)	N4—C6—C5	178.33 (17)
C2—S3—C5	97.88 (7)	S2—C3—H3A	109.00
N2—N1—C1	111.85 (12)	S2—C3—H3B	109.00
N1—N2—C2	112.14 (13)	C4—C3—H3A	109.00
S1—C1—S2	121.11 (9)	C4—C3—H3B	109.00
S1—C1—N1	115.19 (11)	H3A—C3—H3B	108.00
S2—C1—N1	123.63 (11)	S3—C5—H5A	109.00
S1—C2—S3	121.93 (8)	S3—C5—H5B	109.00
S1—C2—N2	114.99 (11)	C6—C5—H5A	109.00
S3—C2—N2	123.07 (11)	C6—C5—H5B	109.00
S2—C3—C4	111.14 (10)	H5A—C5—H5B	108.00
N3—C4—C3	178.79 (16)		
			
C2—S1—C1—S2	−177.61 (10)	C5—S3—C2—N2	0.62 (14)
C2—S1—C1—N1	−0.51 (12)	C2—S3—C5—C6	−68.78 (12)
C1—S1—C2—S3	179.01 (10)	C1—N1—N2—C2	0.16 (19)
C1—S1—C2—N2	0.61 (12)	N2—N1—C1—S1	0.32 (17)
C3—S2—C1—S1	153.44 (9)	N2—N1—C1—S2	177.33 (11)
C3—S2—C1—N1	−23.41 (15)	N1—N2—C2—S1	−0.57 (17)
C1—S2—C3—C4	−60.50 (12)	N1—N2—C2—S3	−178.96 (11)
C5—S3—C2—S1	−177.66 (9)		

### Hydrogen-bond geometry (Å, º)

**Table:**

*D*—H···*A*	*D*—H	H···*A*	*D*···*A*	*D*—H···*A*
C3—H3*A*···N1^i^	0.99	2.60	3.407 (2)	139
C5—H5*B*···N3^ii^	0.99	2.35	3.267 (2)	153

Symmetry codes: (i) −*x*, −*y*+1, −*z*+1; (ii) −*x*, *y*−1/2, −*z*+1/2.
